# IL-6 as a central driver of immune evasion in PDAC: from IDO-mediated tolerance to multi-pathway immunosuppression

**DOI:** 10.3389/fimmu.2026.1885999

**Published:** 2026-07-07

**Authors:** Joyce Wang, Jolene Su Yi Tan, Vishal G. Shelat, Jackwee Lim

**Affiliations:** 1 Singapore Immunology Network (SIgN), Agency for Science, Technology and Research (ASTAR), Singapore, Singapore; 2 Department of Neurology, Tan Tock Seng Hospital, Singapore, Singapore; 3 Department of General Surgery, Tan Tock Seng Hospital, Singapore, Singapore; 4 Lee Kong Chian School of Medicine, Nanyang Technological University, Singapore, Singapore

**Keywords:** immune checkpoints, immune evasion, JAK-STAT-3 signalling, pancreatic ductal adenocarcinoma, regulatory T cells, targeted therapy, tumour microenvironment

## Abstract

Pancreatic ductal adenocarcinoma (PDAC) is among the most lethal cancers and is characterised by an immunologically “cold” microenvironment that limits responses to immunotherapy. This review focuses on interleukin-6 (IL-6), produced by stromal and immune cells as well as PDAC cells, as a central driver of immune evasion via JAK–STAT3 signalling. We outline how SOCS3 silencing in tumour cells inverts the IL-6–IDO relationship seen in dendritic cells, generating an autocrine IDO–Kyn–AhR–IL-6–STAT3 feedforward loop. We further describe IL-6–mediated stabilisation of PD-L1, sustained PD-1 expression on CD8^+^ T cells, and ST6GAL1-driven hypersialylation that activates Siglec glyco-immune checkpoints. In addition, we examine the IL-6–Blimp-1–IL-10 axis and its reprogramming of dendritic cell and T cell compartments. Given the repeated failure of IL-6(R) blockade in clinical trials, we contrast these outcomes with the more encouraging signals from RAS-targeted strategies. We propose rational IL-6–integrated combinations with upstream oncogenic targeting may represent an unrealised frontier for this disease.

## 1 Introduction

Approximately 95% of patients with pancreatic cancer still do not survive (1), and clinical outcomes have shown little improvement (2) over the last four decades. Key hallmarks of pancreatic ductal adenocarcinoma (PDAC, the most common form of pancreatic cancer) include a dense fibrotic stroma comprising over 80% of tumour mass (3) within a highly immunosuppressive microenvironment comprising immunomodulatory cancer-associated fibroblasts (CAFs) derived largely from activated pancreatic stellate cells (4), and myeloid cells including myeloid-derived suppressor cells (MDSCs) and tumour-associated macrophages (TAMs), which can produce high levels of IL-6 (5–9), and in some settings further promote Tregs proliferation instead of mediating Tregs suppression in highly inflamed tissues (10, 11).

While several cytokines are traditionally known to be upregulated in pancreatic cancer including IL-6, IL-8, IL-10 and TNFα (12), high serum IL-6 level is strongly correlated with PDAC disease progression (13, 14). Presently, many IL-6 reviews cover its broad roles across cancers, inflammatory diseases or autoimmunity (15–17). Here, we provided a more mechanistic view of IL-6-STAT3 pathways in PDAC including how IL-6 has opposite effects on IDO depending on SOCS3 status, integration of glyco-immune checkpoints (ST6GAL1) that can shape a highly immunosuppressive tumour environment, multi-level PD-L1 regulation, the less common IL-6-Blimp-1-IL-10 axis, and highlight clinical lessons and emerging therapeutic strategies to improve PDAC patients’ outcomes.

## 2 Methods

This review is a narrative mechanism-focused synthesis of the literature of IL-6 and its associated pathways in the settings of pancreatic ductal adenocarcinoma. It emphasizes key biological domains, including PDAC therapeutic resistance, IDO-dependent immune plasticity, cell-type specific inversion, control of PD-1 and PD-L1 expression and other broad co-inhibitory receptor programming.

Relevant studies were identified through systematic searches of major biomedical databases including PubMed, Web of Science and ClinicalTrials. The search strategy combined terms describing IL-6 (R) primary and secondary roles in PDAC with terms related to inflammatory and metabolic mechanisms, such as immunometabolism, cell exhaustion, immunosuppression, Indoleamine 2,3-dioxygenase (IDO), PD-1/PD-L1 and Siglecs. Relevant peer-reviewed articles, clinical trials, and foundational papers relating to PDAC were retrieved from year 2004 to 2026 but with greater emphasis for papers published within the last five years. Older studies were included selectively when considered to be foundational or highly relevant to the topic. Reference lists of selected articles were also screened to identify additional relevant publications.

## 3 Pancreatic ductal adenocarcinoma

Pancreatic cancer can originate in either the endocrine or exocrine region, but most commonly arises from the exocrine compartment (18, 19). PDAC accounts for more than 85% of all pancreatic malignancies (20). Due to the lack of symptoms at the early stage of cancer development, PDAC are often diagnosed at a late stage (21), when the cancers would have metastasized to the liver or lymph nodes (22).

PDACs arise from precursor lesions – specifically pancreatic intraepithelial neoplasias (PanINs found in small ductal) and intraductal papillary mucinous neoplasms (IPMNs found in main ductal or its branches) – which are characterized by their precancerous nature and high likelihood of progressing into cancer (23, 24). This progression is commonly associated with stepwise accumulation of tumour-associated genetic mutations (25, 26). KRAS mutations are said to drive PanINs and IPMNs initiation (27), whereas additional mutations in genes such as cyclin-dependent kinase inhibitor 2A (*CDKN2A*) and tumour protein p53 (*TP53*) are crucial for its progression from low-grade to high-grade dysplasia (27).

## 4 The extracellular matrix-IL-6 axis in PDAC therapeutic resistance

**Figure 1:**
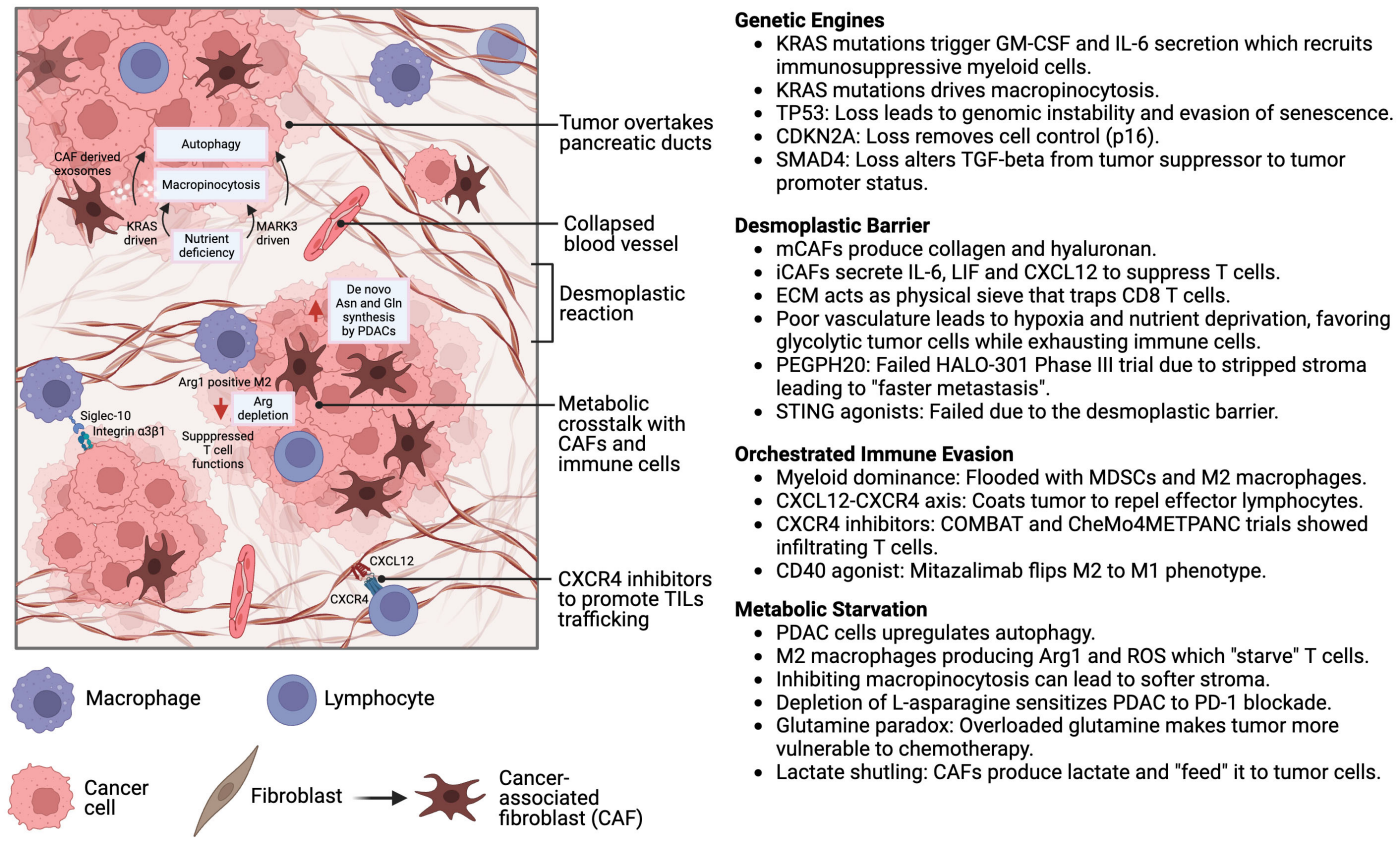
Immune-cold pancreatic ductal adenocarcinoma (PDAC): stromal, metabolic and immune mechanisms driving immune evasion. PDAC is characterized by oncogenic KRAS-driven metabolic rewiring, a dense desmoplastic stroma, and profound immune suppression. cancer-associated fibroblasts (CAFs) deposit collagen and hyaluronan, secrete immunomodulatory cytokines, and create a physical barrier that limits vascular perfusion and T cell infiltration. In parallel, tumour-associated myeloid cells, including macrophages and MDSCs, promote T cell suppression, arginine depletion and immune exclusion, These features collectively generate a hypoxia, nutrient deprived, and immune-cold tumour microenvironment that limits antitumour immunity and contributes to resistance to therapy.

A major contributor to therapeutic resistance in PDAC is the dense desmoplastic extracellular matrix, which serves not only as a physical barrier but also as a biochemical modulator of tumour-stroma communication (**Figure 1**). This fibrotic network restricts chemotherapeutic penetration and impedes immune cell infiltration by limiting T-cell migration and activation (28, 29). Within this complex tumour microenvironment, the major IL-6 producing cells – cancer-associated fibroblasts and tumour-activated myeloid cells – play central and dynamic roles in shaping immunosuppression and therapeutic resistance.

Activated pancreatic stellate cells (PSCs) secrete IL-6 alongside TGF-β and structural extracellular matrix components such as collagen and hyaluronan (30), perpetuating both inflammation and fibrosis. CAFs, which arise from PSC activation and other mesenchymal lineages, represent the major stroma reservoir of IL-6 in PDAC (**Table 1**) (6). Through persistent IL-6 release, these fibroblastic populations stimulate JAK/STAT3 and NF-κB signalling in both tumour and immune cells, fostering chemoresistance to gemcitabine (5, 6, 31). Simultaneously, CAF-driven deposition of collagens, proteoglycans, and hyaluronic acid increases extracellular matrix stiffness and interstitial pressure (4), establishing a feedback loop where mechano-transduction sustains IL-6 production and stroma activation (32). 

**Table 1:** Major mechanistic pathways of IL-6-STAT3 influence across multiple cell types in PDAC.

Cell type	Key pathway(s)	Level of evidence	Potential intervention strategy
PDAC tumour cells and TAMS	• Activated STAT3 stabilizes HIF-1α which drives immunosuppressive TAMs. • SOCS3 methylation inhibits IDO degradation despite presence of IL-6. • Activated STAT3 leads to upregulation of PD-L1. • Activated STAT3 leads to upregulation of ST6GAL1, resulting in glycan-immune inhibition. • KRAS mutation upregulates IL-6 (33), activating STAT3 and promoting PanIN progression.	• PDAC tumour and murine samples (34) • PDAC cell lines and PDAC tumour vs healthy tissue (35) • PDAC cell lines and PDAC tumour vs adjacent tissue (36) • PDAC chemo-resistant and metastatic cell lines (37) • PDAC primary and metastatic tumour patient-derived cell lines and murine models (38, 39)	• STAT3 inhibitors • IL-6/6(R) inhibitors • KRAS inhibitors
CAFs	• Mutations in KRAS and activated STAT3 result in cancer-derived lactate, a signalling molecule which drives IL-6 production.	• PDAC tumour samples (40)	• STAT3 inhibitors • IL-6/6(R) inhibitors • KRAS inhibitors
TAMs and Bregs	• IL-10 from TAMs and Bregs upregulate CTLA-4, TIGIT and 2B4 on T cells. • TGF-β from TAMs and Bregs promotes Treg accumulation.	• PDAC tumour and blood samples (41, 42) • Healthy patients (43)	• STAT3 inhibitors • Immune checkpoint inhibitors
MDSCs	• Activated STAT3 in MDSCs promotes ROS generation, upregulating Arg1 and IDO expression, contributing to T cell anergy (44).	• PDAC spleen samples (44, 45)	• STAT3 inhibitors
Activated PSCs	• TGF-β– NF-κB-IL-6 amplification loop.	• PDAC tumour samples (46)	• STAT3 inhibitors • IL-6/6(R) inhibitors
Immune cells	• STAT3 propagates Rorc activation in Tregs, facilitating Th17-like Tregs conversion. • SOCS3 promotes IDO degradation, generating IDO- immunogenic DCs. • SOCS3 promotes IDO degradation, prevents Kyn production that is needed to activate AhR. • IL-10 from TAMs and Bregs upregulates TIM-3, propagates STAT3 upregulation and increases IL-10 production in cDC2.	• PDAC tumour samples (47) • PDAC spleen samples (45) • PDAC cell lines and murine model (48) • PDAC metastatic cell line conditioned medium effect on DCs (49)	• STAT3 inhibitors • IL-6/6(R) inhibitors • Immune checkpoint inhibitors

The tumour microenvironment profoundly shapes the biology of IL-6 producing cells through hypoxia, nutrient deprivation and immune polarization (**Table 1**). In PDAC, extracellular matrix-driven hypovascularization increases interstitial pressure and exacerbates intratumoural hypoxia, thereby activating hypoxia-inducible factor-1-α (HIF-1α)-dependent transcriptional programmes that sustain IL-6 expression (50). At the same time, these conditions promote the accumulation of immunosuppressive CD206^hi^ and fibrotic TAM states, previously grouped under the broad M2-like category (51, 52). These TAM subsets secrete IL-10 and TGF-β (53), reinforcing cytotoxic immune suppression and consolidating the immunologically cold phenotype of PDAC (54).

Hannifin S et al. has previously shown that hypoxic TAMs are strongly linked to elevated IL-6-STAT3 pathway activity and immunosuppression in both human and mouse PDAC (34). Metabolic constraint further amplifies IL-6-STAT3 activation exacerbated by the desmoplastic barrier and extensively rewires tumour cell metabolism *via* stabilization of HIF-1α (55, 56). HIF-1α upregulates the transcriptional expression of numerous glycolytic enzymes by binding to the hypoxia response element (HRE) consensus sequence (56), resulting in an inefficient but highly glucose-consumptive glycolysis (57, 58). Thus, elevated glycolysis is a metabolic feature and results in reduced glucose availability in PDAC, approximately 50% lower than in benign pancreatic tissue (59). Under oxidative and metabolic stress, particularly in the presence of granulocytic myeloid derived suppressor cells (G-MDSCs), CAFs undergo metabolic rewiring that preserves IL-6 production even under glucose-limited conditions (40, 58, 60). The resulting accumulation of lactate acidifies the PDAC tumour microenvironment, suppressing T-cell and NK-cell effector function while favouring regulatory T-cell (Treg) persistence, as previously reviewed by Gao F et al (61). Together, these metabolic adaptations maintain sustained IL-6 output and reinforce an immune-evasive niche.

Collectively, IL-6 producing CAFs and myeloid cells function as metabolic and inflammatory gatekeepers within the PDAC tumour microenvironment. By integrating cytokine signalling, extracellular matrix remodelling, and metabolic adaption, they sustain an immunosuppressive and therapy-resistant ecosystem that continues to impede treatment responses. Therapeutic stromal remodelling has therefore yield mixed results: in the HALO-301 trial, hyaluronidase-mediated extracellular matrix depletion with pegvorhyaluronidase alfa did not improve outcome and raised concerns that excessive matrix removal may facilitate invasion and metastasis (62). These findings highlight the dual role of the extracellular matrix-IL-6 axis, in which matrix stiffness promotes therapeutic resistance, whereas complete stromal ablation may inadvertently remove constraints on tumour spreading.

Taken together, evidence supporting an extracellular matrix-IL-6 axis in PDAC therapeutic resistance is substantial with direct PDAC data demonstrating CAF- and PSC-derived IL-6 production (40), HIF-1α-dependent upregulation and gemcitabine resistance (direct PDAC evidence shown in *in-vitro*, murine PDAC models and patient-derived) (56, 63). The role of metabolic co-option by granulocytic-MDSCs preserving IL-6 output under glucose deprivation is supported by indirect murine data and requires direct confirmation in PDAC (40). The primary therapeutic implication is that targeting the extracellular matrix alone is insufficient and must be combined with multi-modal IL-6 pathway(s) inhibition to achieve immunological reprogramming. A key unresolved question is whether the sequence of stromal depletion versus IL-6 blockade determines outcome, given the contrasting lessons from HALO-301. 

## 5 Multimodal IL-6-gp130 signalling drives STAT3-dependent immune suppression

**Figure 2:**
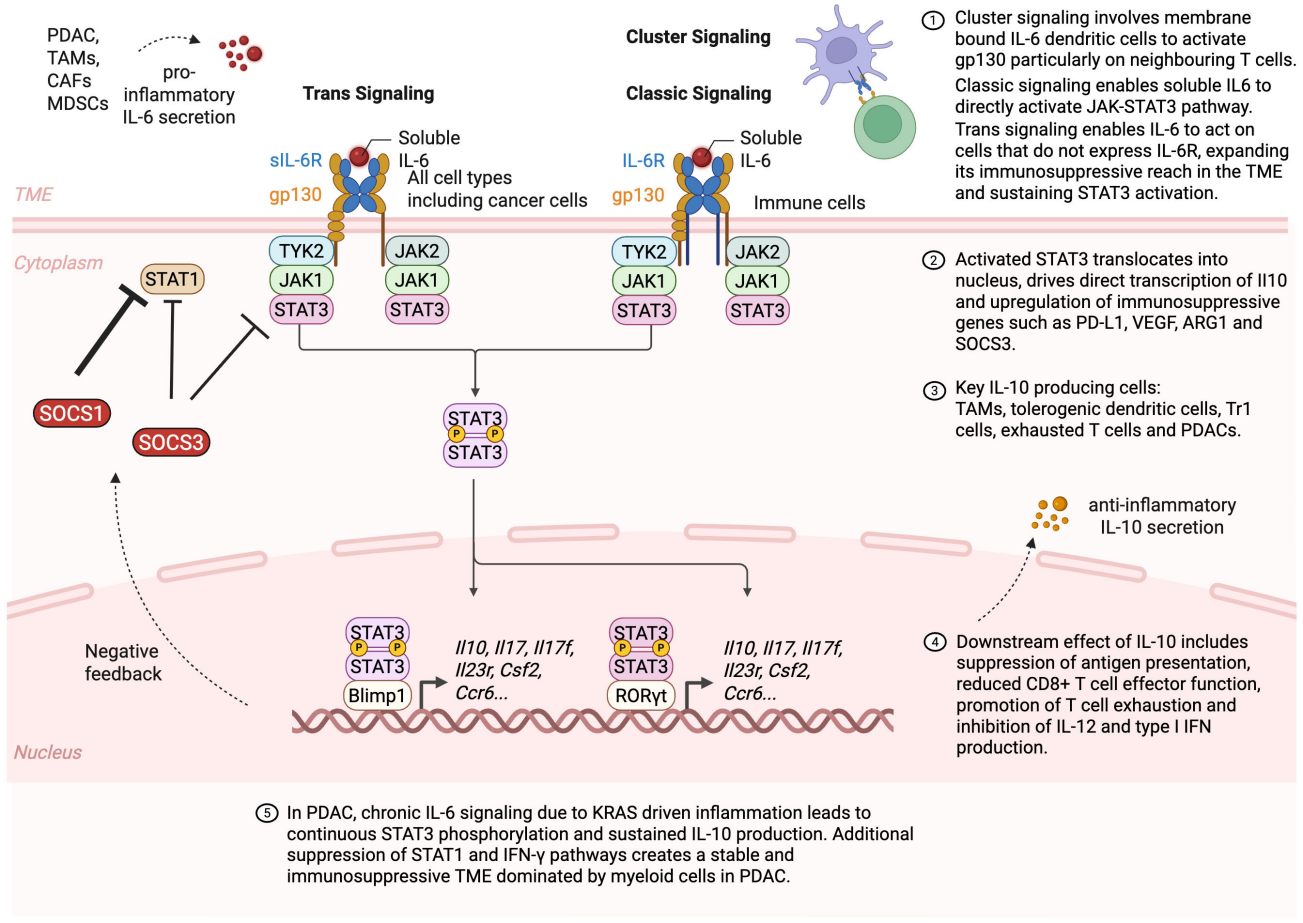
IL-6 signalling pathways shaping immune suppression in PDAC. IL-6 produced by PDAC cells and tumour-associated stromal and myeloid cells signals through classic, trans and cluster modes to activate JAK1/JAK2/TYR2-STAT3 pathways in immune and cancer cells. In classic and trans signalling, IL-6 binding to membrane-bound IL-6R or soluble IL-6R, together with gp130, drives STAT3 phosphorylation, nuclear translocation and transcription of immunosuppressive genes, including IL10, VEGF, ARG1 and SOCS3. Cluster signalling further amplifies local immune activation through membrane-bound Il-6 on dendritic cells and neighbouring T cells. Sustained IL-6-STAT3 signalling promotes IL-10 production by TAMs, tolerogenic dendritic cells, Tr1 cells, exhausted T cells and PDAC cells, while negative feedback via SOCS1 and SOCS3 modulates pathway intensity. Collectively, chronic IL-6 signalling reinforces an immunosuppressive, STAT3-dominant tumour microenvironment that supports PDAC progression and immune evasion.

IL-6 and its receptor components are expressed across multiple immune cell subsets and are markedly upregulated in human PDAC (25), where their expression correlates with reduced overall survival (64). IL-6 signalling is initiated by the formation of a hexameric receptor complex composed of two IL-6 molecules, two ligand binding α-subunits (IL-6R), and two signal-transducing β-subunits (glycoprotein 130, gp130). This complex mediates three mechanistically distinct signalling modes: classic-, trans- and cluster signalling (**Figure 2**).

In classic signalling, IL-6 engages membrane-bound IL-6R (mIL-6R) on a restricted set of cells, promoting gp130 recruitment and homodimerization. These confine signalling to IL-6R expressing cell populations such as hepatocytes and select leukocyte subsets (25, 65). In contrast, trans-signalling occurs when IL-6 binds soluble IL-6R (sIL-6R), generating a complex capable of activating gp130 on virtually all cells, as gp130 is ubiquitously expressed. This mechanism substantially expands the spectrum of IL-6 responsive cells in PDAC, enabling modulation of stroma, endothelial, and immune compartments that lack mIL-6R. Soluble IL-6R is primarily generated through proteolytic shedding of mIL-6R by the metalloproteases ADAM10 and ADAM17, with a lesser contribution from alternatively spliced IL-6R mRNA (65). 

A third mode, termed cluster signalling, involves the presentation of membrane-bound IL-6-IL-6R complexes on “transmitter” cells, such as SIRPα^+^ DCs, to gp130 on neighbouring “receiver” T cells during cognate interaction (66). This configuration promotes spatially restricted gp130 clustering at the immune synapse, facilitating localized IL-6 signalling that directly couples a dendritic cell bound IL-6Rα to T-cell, leading to differentiation of Th17 subset (66).

Activation of gp130 triggers downstream signalling via associated JAK kinases, leading to phosphorylation and dimerization of STAT transcription factors, alongside activation of MAPK and P13K pathways. Persistent IL-6-gp130 signalling – particularly through STAT3, MAPK, and PI3K axes - drives transcriptional programmes that promote inflammation, tumour cell survival, invasion, and immune suppression, thereby contributing to PDAC progression (25).

Negative regulation of this pathway is mediated in part by suppressor of cytokine signalling 3 (SOCS3), a STAT3-inducible proteins that binds phosphorylated gp130 to promote ubiquitin-dependent receptor degradation. SOCS3 can also directly inhibit JAK1/2, thereby attenuating downstream STAT activation (67).

## 6 IL-6- and the TGF-β control of Treg/Th17-like Treg balance in PDAC

IL-6 sits at the centre of a reinforcing inflammatory circuit in PDAC, integrating cues from non-canonical TGF-β signalling, Th17 biology and stromal activation. Non-canonical TGF-β signalling, particularly via NF-κB–dependent pathways, can promote pro-inflammatory cytokine production, including IL-6 by macrophages and monocytes; consistent with this, the IL-6 promoter contains putative NF-κB binding sites (68, 69). In activated human PSCs, neutralization of either IL-6 or TGF-β attenuates the corresponding downstream signalling pathway, supporting the existence of a reciprocal autocrine TGF-β–IL-6 loop (70).

**Figure 3:**
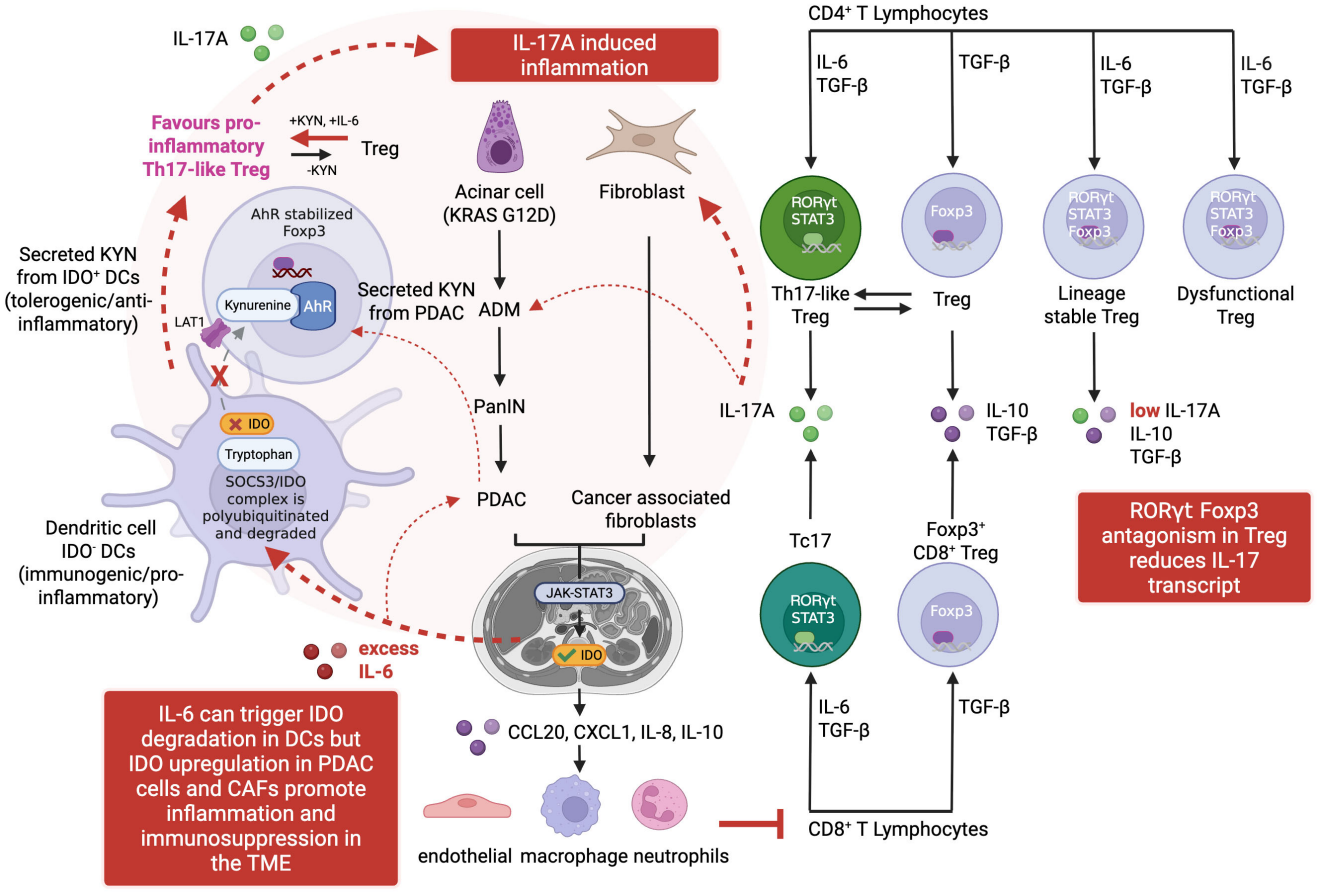
IL-6 driven interplay among IDO-dependent immune plasticity, inflammation and immunosuppression in PDAC. IL-6 produced in excess within the tumour microenvironment orchestrates a complex network of pro-tumourigenic signal across multiple cell types. In dendritic cells, excess IL-6 triggers IDO degradation while paradoxically upregulating IDO in PDAC cells and CAFs via JAK-STAT3 signalling, promoting concurrent inflammation and immune evasion. In the absence of IDO in dendritic cells, PDAC cells themselves secrete kynurenine which shifts kynurenine metabolism towards AhR-mediated Foxp3 stabilization, favouring conversion of conventional Tregs towards a pro-inflammatory Th17-like Treg phenotype. KRAS G12D-driven acinar-to-ductal metaplasia and subsequent PanIN progression to PDAC, alongside fibroblast activation into CAFs, amplify IL-17A induced inflammation. In the adaptive immune compartment, combined IL-6 and TGF-β signalling from CD4^+^ T cells drives differentiation into functionally distinct Treg subsets-characterized by varying expression of RORγt, STAT3 and Foxp3. Collectively, these mechanisms illustrate how IL-6 functions as a central hub coordinating immunosuppression and tumour-promoting inflammation in PDAC. IDO, indoleamine 2,3-dioxygenase; KYN, kynurenine; ADM, acinar-to-ductal metaplasia; PanIN, pancreatic intraepithelial neoplasia; AhR, aryl hydrocarbon receptor.

IL-6 largely influences CD4^+^ T cell fate (**Figure 3**). Naïve CD4^+^ T cells can differentiate into either Foxp3^+^ regulatory T cells (Tregs), which restrain immune responses, Th17 cells, that are pro-inflammatory and driven by RORγt. TGF-β alone favours Treg differentiation, whereas TGF-β together with IL-6 classical signalling promotes Th17 polarization (66, 71). In this context, IL-6 receptor (IL-6R) signalling shifts the balance away from immune regulation and towards inflammation, thereby shaping the tumour microenvironment (66). 

At the transcriptional level, IL-6 dependent STAT3 signalling and IL-2 dependent STAT5 signalling exert opposing effects on the Foxp3-RORγt axis. STAT3 activates Rorc transcription and promotes RORγt expression, whereas STAT5 supports Foxp3 expression through binding to the Foxp3 conserved non-coding sequence 2 (CNS2) and enhances IL-10 production. Although Foxp3 normally suppresses RORγt-mediated IL-17 transcription, sustained STAT3 activity can weaken this restraint and permit the emergence of hybrid Foxp3^+^RORγt^+^IL-17^+^ Tregs (Th17-like Tregs). These cells retain Treg lineage identity yet acquire inflammatory function, reflecting the plasticity of the Treg compartment under persistent IL-6 driven signalling (72, 73).

This balance has relevance in PDAC, where hybridized RORγt+ Tregs are increased and Foxp3-only Tregs are reduced relative to healthy donors (73). Such hybrid Tregs are immunologically ambivalent: they suppress anti-tumour T cell responses while also supporting protumour inflammation (47). More broadly, IL-17 produced by the hybrid Th17-like Tregs, conventional Th17 and γδT17 cells contributes to pancreatic acinar-to-ductal metaplasia and early PanIN progression through IL-17RA-dependent signalling in dysplastic epithelium (74, 75). IL-17 further amplifies immunosuppression by promoting MDSCs, tumour associated neutrophils, Treg maturation and CAF activation (76).

IL-17 also acts upstream of IL-6 production. Tc17 cells (IL-17A producing CD8^+^ T cells) can induce iCAF-associated gene expression through TNF and IL-17A synergy, driving the conversion of resident PSCs into inflammatory CAFs (77, 78), a major source of IL-6 in PDAC. Thus, IL-17 functions as an amplifier of IL-6-rich stromal inflammation. In turn, IL-6R-STAT3 signalling reinforces RORγt expression and IL-17 production in Th17-like Tregs, establishing a feed-forward inflammatory loop in which IL-6 occupies a dominant position.

Although IL-6 and IL-17 are classically viewed as pro-inflammatory cytokines, in PDAC they cooperate to sustain a largely immunologically “cold” tumour microenvironment. Rather than promoting effective anti-tumour immunity, this inflammatory state preferentially expands suppressive immune populations and tumour-supportive stromal programmes, thereby facilitating disease progression.

## 7 IL-6 signalling and the IDO*^+/-^*^deficient^ dendritic cell circuit

### 7.1 The IDO-TGF-β self-amplification loop in IDO^+^ dendritic cells

The metabolic enzyme Indoleamine 2,3-dioxygenase (IDO) catalyses the rate-limiting first step of tryptophan catabolism (79), converting tryptophan into kynurenines (Kyn), and is a critical regulator of peripheral immune tolerance (**Figure 3**). Constitutively expressed at high levels in plasmacytoid dendritic cells (pDCs), IDO shapes the phenotype of neighbouring cell populations, most notably CD4^+^ T cells, in a manner that favours tolerance. Consistent with this, Ruders, JH et al. has reported circulating TGF-β peptide specific CD4^+^ T cell in PDAC patients (80). Central to this tolerogenic programme is TGF-β self-amplification loop (81), whereby TGF-β signalling in dendritic cells activates the Fyn tyrosine kinase, which phosphorylates immunoreceptor tyrosine-based inhibitory motif domains in IDO, creating docking sites for phosphatases SHP-1 and SHP-2. This TGF-β/IDO/SHP signalling axis drives non-canonical nuclear factor-κB (NF-κB) activation through p52/ReIB pathway, in part by suppressing *interleukin-1 receptor-associated* kinase 1 (IRAK1), thereby preventing transcriptional induction of IL-6 (82).

The net result is a shift in DC secretory output away from pro-inflammatory cytokines (82). Production of type I interferons and TGF-β is instead favoured, generating positive feedback that promotes the differentiation of naïve CD4^+^ T cells into Foxp3+ Tregs (81). This self-reinforcing circuit actively suppresses IL-6 production within the tolerogenic niche (83).

### 7.2 IL-6 receptor-SOCS3 results in IDO-deficient dendritic cells

However, IL-6 receptor (IL-6R) signalling counters the IDO circuit through SOCS3, which binds to the immunoreceptor tyrosine-based inhibitory motifs (ITIMS) of IDO protein, and recruits E3 ubiquitin ligase complex for proteasomal degradation (84). By collapsing IDO protein abundance in DCs, this results in immunogenic IDO-deficient DCs which are less capable of secreting Kyn, that would favour Th17-like Tregs (81, 84).

The phenotypic plasticity of Tregs and Th17-like Tregs has been reported in PDAC patients (47, 81, 85). Environmental cytokine cues, particularly the balance between TGF-β and IL-6, profoundly influence interconversion between these subtypes. Reduced Kyn secreted from IDO-deficient DCs present a fundamentally altered antigenic and cytokine landscape that can mediate lineage conversion of Tregs to Th17-like Treg cells (83).

Hence IL-6R signalling promotes IDO degradation in dendritic cells via SOCS3, reducing Kyn secretion. This shifts the main Kyn-producing cell types to PDAC (see section 8.1), which plays a critical role in the balance and plasticity of CD4^+^ T cells.

### 7.3 Epigenetic maintenance of Foxp3 lineage commitment

Stable Foxp3 expression is the defining feature of committed Tregs and is maintained by epigenetic mechanisms centred on the conserved non-coding sequence 2 (CNS2), also termed the Treg-specific demethylated region (TSDR), within the Foxp3 locus (86–89). Demethylation of CNS2 is required for stable, heritable Foxp3 expression independent of exogenous TGF-β. In comparison, TGF-β-dependent STAT5 activation facilitates the initial chromatin opening at methylated CNS2, inducing Foxp3 expression (90). Accordingly, abrogation of IL-2R signalling or loss of STAT5 activity is sufficient to destabilize Foxp3 expression (91).

Beyond STAT5-mediated chromatin access, TGF-β signalling promotes the proteasomal degradation of ubiquitin-like, containing PHD and RING finger domains 1 (Uhrf1), a critical epigenetic regulator of DNA methylation maintenance (92, 93). Loss of Uhrf1 triggers rapid passive demethylation of CNS2, providing a direct mechanism by which TGF-β stabilizes Foxp3 expression at the epigenetic level (92). The kynurenine pathway adds a further layer: Kyn, the principal product of IDO-mediated tryptophan catabolism, binds and stabilizes AhR in Tregs (94), and AhR protein complex directly induces Foxp3 transcription while enhancing chromatin accessibility at the Foxp3 locus (95).

### 7.4 IL-6 STAT3 antagonizes the TGF-β-STAT5-AhR axis to destabilize Foxp3

IL-6-STAT3 competes with TGF-β-STAT5 for transcriptional control of Foxp3, TGF-β stabilizes Foxp3 expression through STAT5 activation and cooperative binding with CREB and AP-1 at the CNS2 locus of the Foxp3 gene (90). IL-6 driven STAT3 directly antagonizes this by competing with STAT5 for overlapping binding sites across the *Il17* genetic locus (96)— STAT3 activation suppresses STAT5-dependent Foxp3 transcription, effectively undermining the transcriptional reinforcement that maintains Treg identity.

SOCS3 creates a secondary antagonism. IL-6-STAT3 upregulates SOCS3, which suppresses STAT5 signalling further and drives proteasomal degradation of IDO (84). SOCS3-mediated IDO collapse in DCs reduces both Kyn availability in the TME and AhR activation in Tregs, whereby AhR signalling is a known co-stabilizer of Foxp3 through epigenetic mechanisms, so its loss, downstream of IL-6 removes a second reinforcing input to Foxp3 stability. As Foxp3 protein levels and stability drop, the cells lose their suppressive functions and begin upregulating Th17-associated markers (e.g., IL-17A, CCR6, and RORγt) to become Th17-like Tregs (97).

Through these multi-layered mechanisms operating between DCs and T cells, Foxp3 stability can be reduced to trigger lineage transformation of Tregs to Th17-like Tregs (84, 98, 99).

### 7.5 AhR as an immune checkpoint: intersection with IL-6 driven IDO collapse

AhR knockout in PDAC cells has been found to promote MHC-I expression (48). Thus, AhR has emerged as a candidate immune checkpoint receptor, exhibiting significant positive co-expression with inhibitory receptors including CD200R1, CD47, PD-1, and TIM-3), as well as inhibitory ligands (BTLA, PD-L1, and PD-L2) (100). Active AhR signalling in DCs and Tregs reinforces suppressive phenotypes and dampens immune activation. IL-6-SOCS3-mediated IDO collapse therefore not only removes Kyn from the extracellular milieu but also functionally inactivates AhR-dependent immune checkpoint programming, converting IDO^+^ tolerogenic DCs to an immunogenic IDO-deficient state, as strong antigen-presenting cells capable of licensing effector T cell responses.

## 8 The paradox of IL-6 and IDO in PDAC: cell-type specific inversion

### 8.1 Autocrine IL-6-IDO co-amplification loop in tumour cells

**Figure 4:**
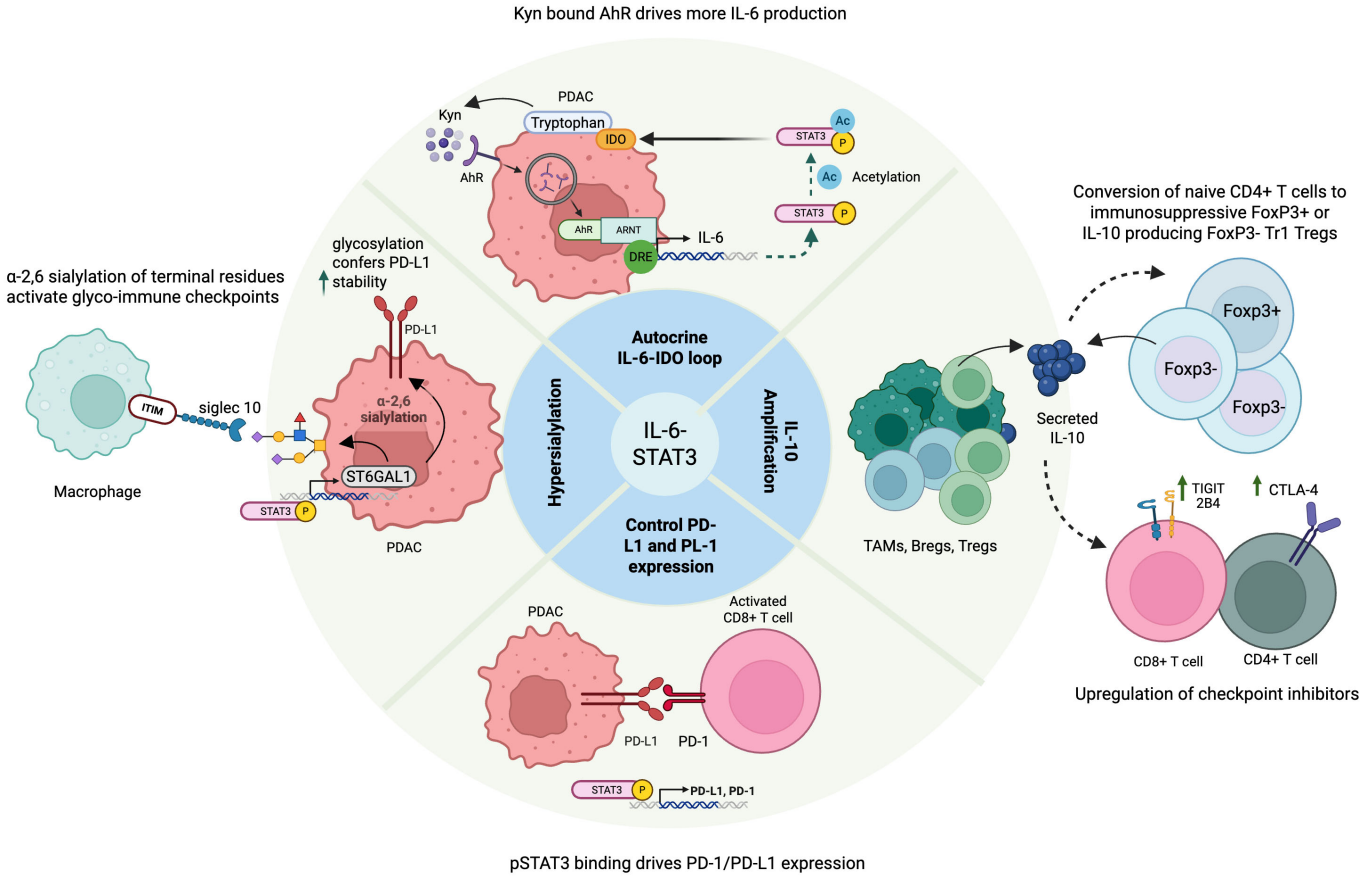
IL-6-STAT3-mediated immunosuppressive pathways in pancreatic ductal adenocarcinoma (PDAC). IL-6 signals through STAT3 to orchestrate four interconnected immunosuppressive mechanisms in the tumour microenvironment (1). Autocrine IL-6-IDO loop (top): In PDAC cells, phosphorylated STAT3 acetylation enhances IDO expression, driving tryptophan catabolism to kynurenine (Kyn). Kyn binds the aryl hydrocarbon receptor (AhR), which heterodimerizes with ARNT and binds dioxin response elements (DRE) to transcriptionally upregulate IL-6, creating a self-amplifying autocrine loop (2). Hypersialylation (left): pSTAT3 transcriptionally induces ST6GAL1, promoting α-2,6 sialylation of terminal residues on PD-L1, conferring glycoprotein stability. Hypersialylated PD-L1 engages Siglec-10 on tumour-associated macrophages via ITIM-mediated inhibitory signalling, activating glyco-immune checkpoints (3). Control of PD-L1/PD-1 expression (bottom): pSTAT3 directly binds promoter elements to transcriptionally upregulate PD-L1 on PDAC cells and PD-1 on activated CD8^+^ T cells, suppressing cytotoxic T cell activity (4). IL-10 amplification (right): IL-6-STAT3 signalling expands tumour-associated macrophages (TAMs), regulatory B cells (Bregs), and regulatory T cells (Tregs), which secrete IL-10 to convert naïve CD4^+^ T cells into immunosuppressive Foxp3+ Tregs or IL-10-producing Foxp3− Tr1 cells. IL-10 further upregulates checkpoint inhibitors TIGIT, 2B4, and CTLA-4 on CD8^+^ and CD4^+^ T cells, collectively dampening anti-tumour immunity. Together, these pathways position IL-6-STAT3 signalling as a central hub of immune evasion in PDAC.

Anu RI et al. has previously reviewed the immunomodulatory roles of IDO in PDAC (101). The impact of IL-6 on IDO in tumour cells is markedly distinct from its effect in DCs (see sections 7.2 and 8.2), representing a paradigmatic example of context-dependent cytokine signalling. In PDAC, IDO expression was found to be significantly elevated, and it correlated to a poor prognosis (35). Mechanistically, in tumours, IDO activity generates Kyn, which activates AhR and permits its nuclear translocation. In the nucleus, AhR dimerizes with aryl hydrocarbon receptor nuclear translocator (ARNT) and binds dioxin-responsive elements (DRE) in the promoter of target genes, including the IL-6 gene itself, thereby transcriptionally inducing IL-6 expression (**Figure 4**).

IL-6 produced in this manner activates STAT3, which undergoes both phosphorylation and acetylation, and activated STAT3 in turn drives further IDO expression. This creates a fully autocrine and self-reinforcing IDO-Kyn-AhR-IL-6-STAT3-IDO feedforward loop in tumour cells (102). Compounding this circuit, persistent and high-amplitude IL-6 signalling sustains chronic STAT3 activation, which itself suppresses SOCS3 expression, removing the principal brake on this loop (103). The downstream consequence is progressive tryptophan depletion in the tumour microenvironment, which impairs cytotoxic T cell proliferation and function as IDO catalyses the rate-limiting step of tryptophan catabolism.

### 8.2 Inversion of IL-6-IDO polarity by SOCS3 silencing in PDAC tumour cells

The cell-type specific inversion in IL-6-IDO biology between DCs and tumour cells is explained in large part by the epigenetic silencing of SOCS3 in cancer cells. In PDAC, SOCS3 is frequently subjected to hypermethylation and transcriptional silencing (104, 105), thus abolishing the canonical SOCS3-dependent IDO degradation. This is unlike SOCS3 in DCs, which drives IDO degradation (see section 7.2). In this epigenetic context, IL-6 no longer depends on the SOCS3 brake to suppress IDO, and instead operates exclusively through STAT3 to reinforce it, creating the autocrine amplification loop described above. Together, these opposing cell-type specific outcomes illustrate a PDAC paradox in which the presence or absence of functional SOCS3 acts a molecular switch deciding whether IL-6 dismantles or amplifies IDO-dependent immunosuppression.

## 9 IL-6-STAT3 control of PD-L1 and PD-1 expression

### 9.1 Multi-level transcriptional regulation of PD-L1 by IL-6

The immune checkpoint molecules PD-1 and PD-L1 form a central axis of adaptive immunosuppression, inhibiting effector T cell activation while simultaneously reinforcing the immunosuppressive capacity of Tregs (106). Transcriptional and post-transcription control of PD-L1 is highly complex in cancer, with multiple transcription factors — including STAT3, STAT1, c-Jun, HIFs, or NF-κB — binding discrete regulatory elements in the CD274 (PD-L1) gene promoter to induce its expression in tumours (36)and stromal fibroblasts (107, 108). Li et al. has reported that STAT3 can also directly stabilize PD-L1 by preventing ubiquitination and degradation in PDAC (**Figure 4**) (36).

Both IL-6 and IL-10 signalling activates STAT3 downstream that can strongly induce PD-L1, and these effects are additive in certain cell types, particularly macrophages (109). IL-6 further amplifies this programme by triggering IL-10 production in MDSCs, regulatory B (110), creating a cytokine relay that broadens and sustains STAT3-driven PD-L1 induction across multiple cellular compartments of the tumour microenvironment.

### 9.2 Post-translational stabilization of PD-L1 by JAK1-mediated glycosylation

Beyond transcript-level control, IL-6-activated JAK1 phosphorylates Tyr112 of PD-L1 protein. This phosphorylation events recruits the endoplasmic reticulum-associated N-glycosyltransferase STT3A, which catalyses PD-L1 glycosylation and stabilizes the protein against proteasomal degradation (111, 112). Glycosylated PD-L1 is substantially more stable and displays enhanced surface expression, thereby amplifying checkpoint suppression independently of transcriptional induction. This dual transcriptional-post-translational mechanism ensures robust PD-L1 accumulation in the IL-6 rich PDAC microenvironment (**Figure 4**). IL-17, produced by Th17-like Tregs cells arising from IL-6 driven Treg reprogramming, has also been observed to upregulate PD-L1 expression through a mechanism that remains to be fully delineated (113), potentially extending the immunosuppressive reach of IL-6 signalling network.

### 9.3 STAT3-mediated prolongation of PD-1 expression on CD8^+^ T cells

The IL-6-STAT3 axis also targets PD-1 expression on cytotoxic T cells. During cognate T cell receptor engagement, IL-6-dependent STAT3 binds directly to regulatory elements of *pdcd1* (encoding PD-1), prolonging PD-1 expression on activated CD8^+^ T cells (114) (**Figure 4**). This sustains PD-1-mediated inhibitory signalling, thus contributing to T cell exhaustion in the chronic inflammatory milieu of PDAC. In this manner, IL-6 simultaneously induces the ligand (PD-L1) on tumour and stromal cells and prolongs receptor (PD-1) expression on effector T cells, reinforcing checkpoint suppression at both ends of the PD-L1-PD-1 axis.

## 10 IL-6-STAT3-ST6GAL1 mediated sialylation and glyco-immune checkpoints

### 10.1 ST6GAL1: IL-6-driven sialylation as immune evasion

Aberrant tumour hypersialylation has emerged as a glyco-immune checkpoint mechanism, enabling cancer cells to evade immune responses by engaging inhibitory receptors on immune effector cells (115). Sialic acid–binding immunoglobulin-like lectins (Siglecs), expressed on T cells, DCs, macrophages, and B cells mediate this effect (116, 117). These Siglecs signal through immunoreceptor tyrosine-based inhibitory or switch motifs (ITIM/ITSM), leading to suppressed cytotoxic activity of CD8^+^ T cells and NK cells, impaired DC maturation and T cell priming, and favour Treg induction and TAMs polarization (118).

In PDAC, IL-6, drives ST6GAL1 overexpression *via* STAT3, binding to the P3 promoter of the sialytransferase gene (**Figure 4**) (37). ST6GAL1 is a critical enzyme for adding sialic acid residues to terminal N-glycosylated proteins via α2-6-linkage, fundamentally altering glycoprotein structure and function (37, 119). ST6GAL1-mediated sialylation generates sialoglycans recognised by Siglec-2, -3, -9 and -10 (120), and elevated α2,3- and α2,6- sialylation was among the dominant glycomic modifications in PDAC patients (121).

### 10.2 Siglec-mediated suppression of TAMs and tolerogenic antigen presentation

Recently, Saini et al. reported that Siglec-10 expressed on TAMs engages its sialoglycoprotein ligand α3β1 on PDAC cells, which suppresses macrophage-mediated phagocytosis, and thereby promotes immune evasion (122). Another consequential aspect of IL-6 driven hypersialylation is its impact on antigen presentation. Perdicchio et al. reported that sialylated tumour antigens taken up by DCs endows a tolerogenic response, inducing *de novo* Tregs while dampening T effector expansion and IFN-γ production (123).This mechanism directly links IL-6 driven ST6GAL1 activity to antigen specific tolerance in the PDAC tumour microenvironment, a glycoimmune barrier that reinforces resistance to immunotherapies by promoting Sia-antigen-specific tolerance.

## 11 IL-6–Blimp-1–IL-10 axis and broad co-inhibitory receptor programming

### 11.1 STAT3-driven Breg induction and IL-10 amplification

Under the quiescent conditions, B cells produce minimal IL-10 (124). However, STAT3 signalling induces IL-10-competent regulatory B (Breg) programme through cooperation with IRF4 and PRDM1/Blimp-1–associated transcriptional networks. Breg are the most prominent B cell subset identified in PDAC (124, 125), and exert broad immunosuppressive effects not through lineage identity but through secretion of anti-inflammatory cytokines including IL-10, TGF-β and IL-35. These mediators directly sustain MDSCs and TAMs (125, 126), and Bregs can expand Treg populations or converting naive T cells into Foxp3+ Tregs or IL-10 producing Foxp3- Tr1 regulatory cells (127–129).Supporting a role for Tr1-like cells in PDAC, tumour-associated DCs have been shown to induce CD4^+^ T cells expressing Tr1-associated markers including CD39, CD49b, IL-10, and AhR within the pancreatic tumour microenvironment, and overall creating an immunosuppressive environment (130).

IL-6 additionally triggers IL-10 production in MDSCs and other myeloid cells, creating a broader cytokine relay that sustains STAT3 activity across multiple cell types. In macrophages, IL-10 downregulates co-stimulatory molecules CD86, ICAM1, and MHC class II expression, via induction of the E3 ubiquitin ligase March-I (131–133), directly limiting the capacity for effective antigen presentation and T cell priming. Beyond antigen presentation, IL-10 signalling promotes CTLA-4 expression on CD4^+^ T cells and TIGIT and 2B4 (CD244) on CD8^+^ T cells (134). Consistent with this immunosuppressive phenotype, PDAC-infiltrating T cells exhibit elevated expression of TIGIT (41), and high soluble CTLA-4 levels are associated with advanced PDAC severity (42). CTLA-4 outcompetes with costimulatory receptor CD28 for the shared ligands CD80 and 86, for which it has a higher affinity and avidity, thereby attenuating further T cell activation (135). Collectively, IL-10 driven upregulation of CTLA-4, TIGIT and 2B4 compounds the PD-1 exhaustion programme driven directly by IL-6-STAT3, blanketing cytotoxic T cells with multiple co-inhibitory signals.

### 11.2 TIM-3-STAT3 feedforward loop in cDC2-like immunosuppressive dendritic cells

Chronic inflammatory signalling through IL-6 and IL-10 reprograms tumour-infiltrating DCs towards a cDC2-like immunosuppressive phenotype. TIM-3, a co-inhibitory receptor expressed on this DC subset as well as Foxp3+ Tregs (136), reinforces STAT3 activation in reprogrammed DCs, which drives further IL-10 production. IL-10 in turn signals through c-Src to activate the transcription factors Ets1, Ets2, USF1, and USF2, propagating TIM-3 expression and establishing a TIM-3-STAT3-IL-10 positive feedback loop (137). These cDC2-like DCs skew T cell differentiation towards Th2- and Treg responses, further entrenching the immunosuppressive tumour microenvironment (137).

### 11.3 IL-6-mediated impairment of DC differentiation and the cDC2 enrichment paradox in PDAC

Elevated IL-6 within the PDAC tumour microenvironment contributes to defective dendritic cell differentiation and function through aberrant STAT3 activation, thereby suppressing DC maturation (49). In PDAC patients, cDC2s are the predominant infiltrating DC subtype, yet this population is markedly depleted relative to healthy tissue and displays reduced expression of co-stimulation receptors (138–140). This creates a paradox of relative cDC2 enrichment and overall DC scarcity and functional impairment: the remaining DC pool is disproportionately composed of functionally compromised cDC2-like subset, which is then further reprogrammed by the IL-6-IL-10 axis toward immunosuppressive phenotypes (141). The new consequence is a profound distortion of the antigen-presenting cell landscape, with vast downstream implications for CD4^+^ and CD8^+^ T cell differentiation, effector function, and exhaustion within the PDAC tumour microenvironment. 

## 12 Clinical translation of IL-6 pathway targeting: Lessons from the trial landscape

### 12.1 IL-6(R) blockade in PDAC: a pattern of clinical failure

Despite the mechanistic rationale described above, direct targeting of IL-6 or its receptor in PDAC has not translated into meaningful clinical benefits. Two prospective trials have tested IL-6R or IL-6 blockade in combination with immune checkpoint inhibitors in this disease (**Table 2**), and neither demonstrated the efficacy required for continued development.

**Table 2:** Clinical trials of IL-6(R) pathway targeting in PDAC versus RAS-directed strategies.

Study/ref	Population	Intervention	Targets	Key outcomes	Conclusion
Phase Ib/II (142)	Late-stage/metastatic PDAC (n = 31)	Siltuximab, Spartalizumab	IL-6, PD-1	Low dose: • Median progression-free survival (PFS): 1.9 months Median overall survival (OS): 9.3 months• High dose: • Median PFS: 2.1 • Median OS: 3.1	Absence of measurable tumour regression in treated patients. Cannot progress due to no meaningful clinical activity.
Phase II trial ‘TRIPLE-R’ (143)	Refractory pancreatic cancer (n = 26)	Radiotherapy, Ipilimumab, Nivolumab, Tocilizumab	IL-6R, CTLA-4, PD-1	• Median PFS: 1.6 months • Median OS: 5.3 months	Did not meet the criteria for the study to expand and continue.
Phase III RASolute 302 (144)	Previously treated Metastatic PDAC (n = 500)	Daraxonrasib	Multiple RAS variants	• Median OS of 13.2 months • Median PFS: undisclosed	Met all primary and key secondary endpoints.
Interim Phase 1/2 (AMPLIFY-7P) (145)	mKRAS-driven PDAC (n = 158)	Peptides: ELI-002 7P	mKRAS	• Median recurrence-free survival of 16.33 months • Median OS: 28.94 months	*Disease-free survival analysis to be released in 2026.*

In a Phase Ib/II trial combining the anti-IL-6 antibody siltuximab with the anti-PD-1 antibody spartalizumab in patients with late-stage or metastatic PDAC, no measurable tumour regression was observed across either dose cohort. The low-dose cohort achieved a median progression-free survival (PFS) of 1.9 months and a median overall survival (OS) of 9.3 months; the high-dose cohort fared worse, with a median PFS of 2.1 months and a markedly reduced median OS of 3.1 months. The absence of meaningful clinical activity precluded further trial progression.

The Phase II ‘TRIPLE-R’ trial evaluated a more aggressive quadruplet regimen of radiotherapy combined with ipilimumab (anti-CTLA-4), nivolumab (anti-PD-1) and tocilizumab (anti-IL-6R) in patients with progressive pancreatic cancer. Despite multi-modal checkpoint co-inhibition, the trial failed to meet its pre-specified expansion criteria, recording a median PFS of 1.6 months and median OS of 5.3 months. Together, these outcomes highlight a consistent pattern in which IL-6R blockade, even combined with established immune checkpoint inhibitors, fails to reprogram the immunosuppressive PDAC tumour microenvironment at clinically advanced disease stages.

### 12.2 Redefining the therapeutic horizon: RAS-directed strategies in PDAC

The repeated failure of IL-6(R) blockade in PDAC has prompted a re-evaluation of which molecular targets are tractable in this disease. Mounting evidence from two distinct RAS-directed therapeutic strategies—one targeting the oncogenic driver directly at the protein level and one harnessing the immune system against it—offers a compelling mechanistic contrast and potentially transformative clinical results. Daraxonrasib (RMC-6236) is an orally bioavailable, RAS(ON) multi-selective, non-covalent tri-complex inhibitor that targets the active, GTP-bound state of RAS. This modality confers pan-RAS selectivity across the broad spectrum of RAS G12X and other variants present in PDAC—including G12D, G12V, G12R, G12A, G12S and G12C—addressing a limitation that has historically constrained allele-specific inhibitor development in this disease. The clinical impact of this mechanism was demonstrated in the Phase III RASolute 302 trial (NCT06625320). Whereas daraxonrasib targets RAS oncoproteins directly, ELI-002 7P (Elicio Therapeutics) takes an orthogonal immunological approach: activating the patient’s own adaptive immune system against mutant KRAS neoantigens. ELI-002 7P is a seven-valent amphiphile (Amph) cancer vaccine comprising Amph-modified long peptides encoding seven common KRAS/NRAS driver mutations (G12D, G12R, G12V, G12A, G12C, G12S, and G13D) combined with an Amph-modified Toll-like receptor 9 (TLR9) agonist adjuvant (CpG-7909). ELI-002 7P is being evaluated in the Phase 1/2 AMPLIFY-7P trial (NCT05726864) as adjuvant monotherapy in patients with mKRAS-positive PDAC (and other solid tumours). Notably, antigen spreading to personalised tumour neoantigens beyond mKRAS was detected in a subset of patients—a signal suggesting that vaccine-induced T cells are killing tumour cells and releasing new antigens, broadening the immune response beyond the vaccine’s encoded targets.

A mechanistic distinction between these two RAS-directed approaches is noteworthy in the context of this Review. Daraxonrasib acts *upstream* of the immunosuppressive programme described herein — suppressing oncogenic RAS signalling that drives IL-6 production, IDO induction, STAT3 activation, and ST6GAL1 overexpression — and thereby has the potential to dismantle the tumour-intrinsic feedforward loop at its source. ELI-002 7P acts *in parallel*, seeking to generate mKRAS-specific cytotoxic T cells capable of recognising and killing PDAC cells that express these neoantigens. In principle, these strategies are not mutually exclusive: reducing oncogenic RAS activity with daraxonrasib could alleviate T cell exclusion and immune suppression in the tumour microenvironment, while vaccine-induced mKRAS-specific T cells could target residual or resistant tumour clones. The clinical viability of such combinations remains to be tested.

### 12.3 Timing, tumour architecture, and the limits of late-stage IL-6 targeting

IL-6 has a prominent role in pancreatic tumourigenesis, including promotion of inflammatory signalling, myeloid-cell recruitment, impaired dendritic-cell differentiation, STAT3 activation, and early immune tolerance. However, by the time most patients present with advanced or metastatic PDAC, the tumour microenvironment is often already shaped by dense desmoplasia, T cell exclusion, stromal compartmentalisation, and multiple overlapping immunosuppressive pathways. In this setting, IL-6/IL-6R blockade alone is unlikely to fully reprogramme the established stromal–myeloid network or restore effective antitumour immunity. Its value may therefore depend less on broad late-stage use and more on rational combinations, earlier intervention windows, or biologically selected patients.

The absence of biomarker-guided patient selection compounds this challenge. Patients with high circulating IL-6, high intratumoural IL-6 pathway activity, or tumours with demonstrable STAT3-dependent immune suppression may represent more plausible candidates for IL-6-directed therapy, although these markers remain investigational rather than validated predictors. In unselected PDAC, using immune checkpoint inhibition as the therapeutic backbone without first addressing T-cell exclusion, stromal barriers, and myeloid-mediated suppression is likely to limit the efficacy of any added immunomodulatory intervention.

### 12.4 Implications for trial design: earlier intervention, rational combinations, and patient selection

Collectively, these findings suggest that IL-6 blockade may be better suited to earlier disease stages – neoadjuvant or resectable settings – where the tolerogenic programme is still being established and where prevention of immune suppression is mechanistically achievable. Rational multi-modal strategies that simultaneously address stromal barriers (for example, with CXCR4 or TGF-β inhibitors), deplete immunosuppressive myeloid populations, and engage T cell priming alongside IL-6R blockade may more closely match the biological complexity of the PDAC tumour microenvironment. Finally, prospective biomarker stratification — selecting patients with demonstrably IL-6 dependent STAT3 activation, high ST6GAL1 expression, or SOCS3 competent intratumoural DCs — represents an important and as yet untested approach to identifying the subpopulation most likely to benefit from IL-6 pathway inhibition.

To operationalize this approach, we propose a multi-parameter biomarker framework for patient selection in future IL-6 directed trials. Candidate stratification markers include (i) serum IL-6 (>10 pg/mL) as a provisional threshold, to be defined prospectively; (ii) pSTAT3-positive tumour cells by immunohistochemistry (>10% of tumour cells); (iii) SOCS3 promoter methylation status in tumour biopsy (methylation indicating IDO-amplifying PDAC context); (iv) ST6GAL1 protein expression by IHC or RNA *in situ* hybridization; (v) glycosylated PD-L1 as detected by glycan-specific antibody; (vi) Siglec ligand abundance by lectin histochemistry; and (vii) T-cell exclusion score e.g. CD8^+^ T-cell density at tumour margin vs centre. Therefore patients with high IL-6 pathway activation (markers i-iii positive), evidence of glyco-immune checkpoint activity (markers iv-vi), and profound T-cell exclusion (marker vii) represent the stratum for IL-6-STAT3 co-targeting strategies.

## 13 Conclusion

IL-6 is a multi-armed cytokine whose immunosuppressive impact in PDAC extends far beyond any single pathway. Acting primarily through STAT3, and reinforced by IL-10, Kyn–AhR, and JAK1 kinase activity, IL-6 drives a coordinated programme that disrupts tolerogenic DC function, sustains an IDO–IL-6 autocrine loop in SOCS3-silenced tumour cells, stabilizes PD-L1 protein and prolongs PD-1 on exhausted T cells, promotes tumour-surface hypersialylation to engage Siglec-mediated checkpoints, and expands Breg–IL-10 circuits that suppress antigen presentation and co-stimulation. These pathways are interconnected and mutually reinforcing.

Consistent with this biological complexity, earlier clinical trials of IL-6R/IL-6 blockade in PDAC have repeatedly failed to achieve meaningful responses, in contrast to the more encouraging responses reported for RAS-directed strategies. This divergence likely reflects differences in tumour immune architecture and the multiplicity of upstream inputs converging on STAT3 in PDAC. Against this backdrop, RAS-directed strategies in PDAC, including RAS(ON) multi-selective inhibitor daraxonrasib and mKRAS/NRAS peptide vaccines, are generating more promising early clinical signals.

Translating the mechanistic understanding into clinical benefits will require a fundamental shift in trial design strategy, rather than adding IL-6 or IL-6R blockade onto a single immunotherapy backbone, future trials should be designed around the interconnected biology described here: combining IL-6 pathway inhibition with agents that simultaneously dismantle stromal barriers, restore DC function, and counteract glyco-immune checkpoints. The proposed multi-parameter biomarker framework — incorporating serum IL-6, pSTAT3 tumour burden, SOCS3 methylation status, ST6GAL1 expression and T-cell exclusion score — offers a tractable path towards identifying the patient subpopulation most likely to benefit, rather than applying IL-6(R) blockade in an unselected population. The convergence of RAS-directed strategies with IL-6 pathway co-targeting represents a compelling combination hypothesis: as RAS(ON) inhibitors reshape tumour-intrinsic signalling and reduce IL-6 transcription. Realising this potential will depend on prospective biomarker stratification, mechanistically rational combination design, and willingness to test these hypotheses in earlier stage disease where immune intervention has the greatest chance of altering the natural history of PDAC.
